# Towards a mechanistic understanding of carbon stabilization in manganese oxides

**DOI:** 10.1038/ncomms8628

**Published:** 2015-07-21

**Authors:** Karen Johnson, Graham Purvis, Elisa Lopez-Capel, Caroline Peacock, Neil Gray, Thomas Wagner, Christian März, Leon Bowen, Jesus Ojeda, Nina Finlay, Steve Robertson, Fred Worrall, Chris Greenwell

**Affiliations:** 1 School of Engineering and Computing Sciences, Durham University, South Road, Durham DH1 3LE, UK; 2 School of Civil Engineering and Geosciences, Devonshire Walk, Newcastle University, Newcastle upon Tyne NE1 3RE, UK; 3 School of Agriculture, Food and Rural Development, Newcastle University, Newcastle upon Tyne NE1 7RU, UK; 4 Earth Surface Science Institute, School of Earth and Environmental Sciences, University of Leeds, Leeds LS2 9JT, UK; 5 Department of Physics, Durham University, South Road, Durham DH1 3LE, UK; 6 Experimental Techniques Centre, Institute of Materials and Manufacturing, Brunel University, Uxbridge UB8 3PH, UK; 7 Department of Earth Sciences, Durham University, South Road, Durham DH1 3LE, UK

## Abstract

Minerals stabilize organic carbon (OC) in sediments, thereby directly affecting global climate at multiple scales, but how they do it is far from understood. Here we show that manganese oxide (Mn oxide) in a water treatment works filter bed traps dissolved OC as coatings build up in layers around clean sand grains at 3%w/wC. Using spectroscopic and thermogravimetric methods, we identify two main OC fractions. One is thermally refractory (>550 °C) and the other is thermally more labile (<550 °C). We postulate that the thermal stability of the trapped OC is due to carboxylate groups within it bonding to Mn oxide surfaces coupled with physical entrapment within the layers. We identify a significant difference in the nature of the surface-bound OC and bulk OC . We speculate that polymerization reactions may be occurring at depth within the layers. We also propose that these processes must be considered in future studies of OC in natural systems.

## Abstract (web summary)

Minerals are known to stabilize organic carbon in sediments, affecting biogeochemical cycles and global climate, but the mechanism is not understood. Here, the authors suggest that manganese oxides can trap organic carbon and may act as a ‘mineral pump', transforming carbon between labile and refractory forms.

## Graphical abstract

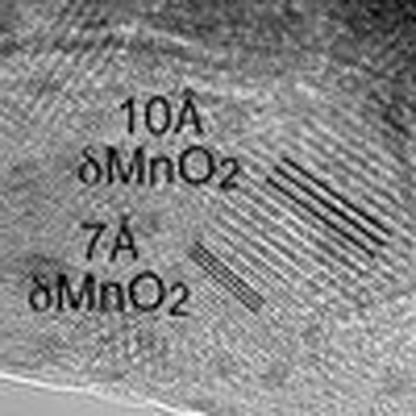

Recent estimates suggest that after rocks (70 million Gt of organic carbon), soils (2,500 Gt) and marine sediments (3,000 Gt) account for the largest reservoirs of organic carbon on Earth^1^,^2^. It is thought that all of this carbon is intimately mixed with minerals^2^. Many researchers have studied the nature of mineral-stabilized organic carbon in soils^3^ and marine sediments^4^, but the majority of these studies are the equivalent of ‘biogeochemical black boxes' where inputs and outputs can be estimated but the internal mechanisms operating in these natural systems are rarely clear due to their inherent complexity. We therefore need to look at simpler, more controlled systems to extract the fundamental mechanistics of mineral–carbon interactions.

Manganese is known to strongly interact with dissolved organic carbon (DOC)^5^,^6^,^7^. Its global role in the stabilization of carbon in sediments, however, is almost entirely unconstrained. Roy *et al.*^8^ recently described the close association between both reactive iron (Fe) and reactive manganese (Mn) with organic carbon (OC) in the marine sediments off the Californian and Oregon coast in the United States. They^8^ found that there was a stronger correlation between Fe and OC compared with Mn and OC and postulated that this was because reactive Mn was more mobile during diagenesis. Lalonde *et al.*^4^ more recently confirmed that Fe-associated OC (Fe-OC) accounts for up to 21% of marine sediment OC, emphasizing the importance of mineral–organic interactions as critical components of the global carbon cycle. However, neither of these studies is able to comment in detail on the specific mechanisms behind the OC stabilization by these redox active metal oxides. This is due to the inherent heterogeneity of natural systems, in terms of microbiology, fluctuating environmental conditions and the mixed mineral assemblages they represent.

To further add to the complexity of studying mineral–OC interactions in natural systems, most OC in sediments is molecularly uncharacterized carbon (MUC)^9^.

Traditionally, OC in sediments has been analysed using acids/alkalis or solvents for extraction and then chromatography for characterization, but these methods are flawed due to the changes induced in the organic carbon during extraction^10^. Thermal gravimetric analysis has been shown to differentiate between pools of organic carbon, broadly defined as ‘thermally labile' (released at temperatures <550 °C) and ‘thermally refractory'^11^,^12^,^13^ (released at temperatures >550 °C). However, both solvent extraction and thermal analysis can suffer from analytical artefacts requiring other, independent analyses. We use X-Ray photoelectron spectroscopy to study the OC bonding environment without either solvent or thermal extraction. Thermally refractory OC in soils is traditionally thought to be composed of black carbon^11^, but it is only an end member in a continuum of thermally refractory OC, as humin is also thermally stable^14^. Black carbon is formed by the incomplete combustion^13^ of organic carbon. Humin (or ‘protokerogen') is thought to be organic carbon that is strongly bound to mineral surfaces^14^. Current theories on how OC becomes partitioned into the thermally refractory humin fraction include selective preservation of refractory molecules, physical protection of OC by mineral occlusion (entrapment of OC within the mineral matrix) and geopolymerization^15^,^16^,^17^.

Geopolymerization describes the oxidative polymerization of low molecular weight monomers to humic substances that can be catalysed by mineral surfaces^17^. Birnessite is one of the most common Mn oxides in terrestrial (for example, in soils^18^ and natural aquatic systems^19^) and marine environments (for example in oceanic ferromanganese nodules^20^,^21^ and in pelagic sediments^21^,^22^). Birnessite also catalyses the polyphenol-Maillard reaction^23^, which generates humic polymers. In contrast, Mn oxides can break down high molecular weight (HMW) humic substances into lower molecular weight (LMW) organic molecules^6^. Whether Mn oxides humify organic molecules into larger HMW molecules or break them down them into smaller LMW molecules is not fully understood but is likely to depend on the balance between oxidants (including oxygen and Fe/Mn oxides) and reductants (such as DOC) in the system^24^. Our study addresses a major gap in understanding about the mechanism of how OC and sediments interact at a molecular and individual mineral scale, invoking both of these aforementioned reaction pathways in the same micro-environment.

In contrast to the ‘black box' approach of studying natural systems, we decided to present experimental data from a more controlled non-natural system. Our data are from a clean water treatment works (WTW) where the main inputs and outputs of Mn and DOC are well constrained. The WTW filter bed consists of birnessite-coated sands (confirmed with extended X-ray absorption fine structure (EXAFS) spectroscopy, data not shown), which represent a far simpler and relatively homogenous mineralogy compared with natural marine sediments or terrestrial soils. Studying this simplified system has significant advantages over natural samples, as we are able to look at the specific chemical mechanisms involved in OC stabilization in concentrated mineral assemblages, largely without biotic interference (see below). This approach enables us to show that the birnessite, one of the most common forms of Mn oxide in marine^20^,^21^,^22^ and terrestrial environments^18^,^19^, builds up in ‘onion shell' like layers in WTWs, trapping DOC at 3%w/w C (0.5%w/w inorganic carbon is also present). We call the trapped organic carbon ‘manganese associated organic carbon' (Mn-OC). We use a novel combination of micro-Fourier transform infrared spectroscopy (FTIR), fluorescence spectroscopy, thermogravimetric analysis (TGA) and X-ray photoelectron spectroscopy (XPS) to analyse this Mn-OC and identify differences between Mn-OC bound at the surface of the Mn oxide coatings (that is, the outermost layer of Mn oxide) and Mn-OC present in the bulk Mn oxide coatings.

## Results

### Sampling

**Figure 1:**
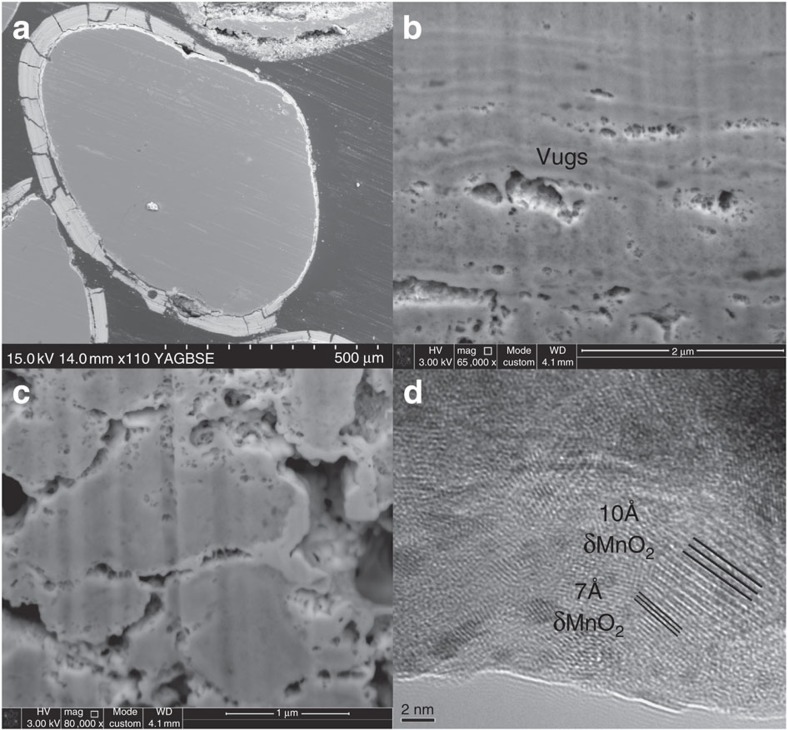
SEM, TEM and FIB images of birnessite coating. (**a**) Scanning electron micrograph showing intact birnessite coating on sand grains. (**b**) Focused ion beam image of birnessite coating showing light and dark laminae at both μm and nm scale. (**c**) FIB image showing close-up of vugs within birnessite coating, apparently partially infilled with lighter (in colour) precipitates. (**d**) Transmission electron micrograph of birnessite coating showing both 7 and 10 Å poorly crystalline birnessite (akin to δMnO_2_). Images **a**–**c** are all in back-scattered mode showing atomic contrast.

The birnessite-coated sand is from a clean water treatment works (WTW) gravity filter bed designed to remove soluble Mn (∼0.2 mg l^−1^) from the influent water (see Methods for further details). The influent water contains natural DOC ∼10 mg l^−1^ with a δ^13^C signature of ∼−27‰ ref. 25 and a small amount of a cationic flocculant (see Methods for further details). The pH is raised to pH 9.2 by flash liming to optimize abiotic manganese removal via precipitation of birnessite as coatings on the sand grains and as Mn oxide particulates in between the grains. Basal respiration is not detectable. It should be noted that not all influent Mn (or DOC) is removed. The filter bed is backwashed on a daily basis to remove particulate Mn oxide to ensure that porosity is retained. The Mn that stays in the filter bed builds up as birnessite coatings (∼100 μm thick) on sand grains (see Fig. 1). We refer to ‘surface bound Mn-OC' as the organic carbon present on the outermost surface of the intact birnessite coatings, while ‘bulk Mn-OC' refers to the organic carbon present throughout the bulk of the birnessite coatings (where bulk material was generated by carefully dislodging the birnessite coatings from the sand grains and sieving to produce the 63-149 μm fraction).

### Surface bound Mn-OC via FTIR and fluorescence spectroscopy

**Figure 2:**
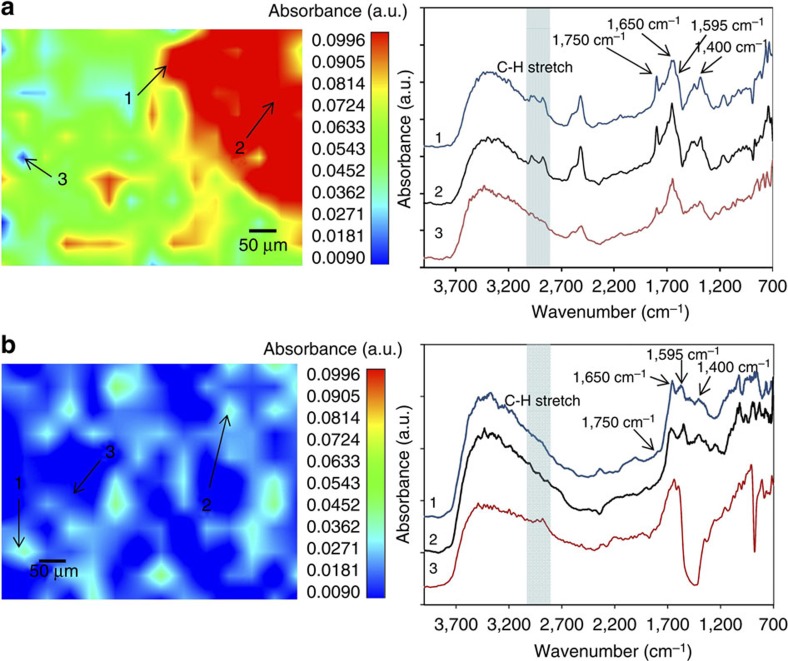
Micro-FTIR images of birnessite coating. (**a**) Micro-FTIR spectroscopy image and spectra of the surface of an intact birnessite coating (washed repeatedly in de-ionized water) showing presence of organic carbon (at three separate points representing low, medium and high OC concentrations). (**b**) Micro-FTIR spectroscopy image and spectra of a (different) surface of an intact birnessite coating (washed repeatedly with 1 M NaOH) showing presence of organic carbon (at 3 separate points representing low, medium and high OC concentrations) at lower concentrations than when washed in de-ionized water. Red-yellow colours show higher concentration of OC and green-blue colours show lower concentration of OC. In the spectra, the blue band shows the C-H stretch between 3,000 and 2,800 cm^−1^ and also labelled are the C=C (1,650 cm^−1^) and carbonyl (1,750 cm^−1^) peaks as well as the ν_sym COO-_ (1,400 cm^−1^) and ν_asym COO-_ (1,595 cm^−1^ in (**a**) and 1,565 cm^−1^ in (**b**)) peaks.

The surface of the intact birnessite coatings was washed repeatedly in de-ionized water (as a control) and in 1 M NaOH (in an attempt to remove surface bound OC) and analysed by micro-FTIR spectroscopy to observe visible Mn-OC (see Fig. 2 where red colours show high OC concentration and blue colours show low OC concentrations) and fluorescence spectroscopy (Table 1) to identify and characterize surface-bound Mn-OC.

The micro-FTIR images show that OC distribution on the surface of the birnessite coating is not homogenous (Fig. 2 shows three spot analyses representing relatively high, medium and low OC concentration in each case). In the micro-FTIR spectra, the pale blue bands highlight molecules adsorbing between 3,000 and 2,800 cm^−1^, which can be attributed to C-H groups^26^. This C-H contribution is less evident for the birnessite that has been washed in NaOH, suggesting that some organics are removed from the birnessite during this wash step. However, the persistence of the broad shoulder between 3,000 and 2,800 cm^−1^ after washing in NaOH indicates that not all the C-H bonds are removed, confirming a strong association between this remaining OC and the birnessite.

The presence of the band around 1,750 cm^−1^ can be assigned to the stretching C=O (ν_C=O_)^27^ and is usually attributed to the presence of carbonyl moieties (such as those found in carboxylic groups, esters, ketones). The band around 1,400 cm^−1^ (ref. 28) can be assigned to the symmetric stretching of C-O of carboxylate groups (ν_sym COO-_), whereas the shoulder corresponding to the anti-symmetric stretching vibration (ν_asym COO-_) is observed at 1,595 cm^−1^ (ref. 29) in the sample washed in de-ionized water and 1,565 cm^−1^ in the sample washed in NaOH. The absorption band around 1,650 cm^−1^ indicates the presence of carbon double bonded to carbon (C=C) such as in alkenes or aromatic rings^30^. Although the peak at 1,750 cm^−1^ (mostly attributable to carboxylic groups) is not readily apparent after washing with NaOH, those at 1,565 and 1,400 cm^−1^ (attributable to carboxylate groups) and those at 1,650 cm^−1^ (attributable to alkenes/aromatic groups) are all apparent in the most concentrated spot analyses of the birnessite washed in NaOH (Fig. 2, bottom, spots 1 and 2). These observations suggest that carbonyl moieties present in carboxylate groups, and alkenes/aromatic groups, form part of the more stable carbon pool that is not removed by NaOH.

Extensive studies made on metal complexes of carboxylic acids have established an empirical correlation between the position of the symmetric stretching (*v*_sym COO_-) and asymmetric stretching (*v*_asym COO_-) of carboxylate groups and the difference in frequency between them (Δ*v*)^31^,^32^,^33^,^34^,^35^. In our case, as can be seen in Fig. 2, Δ*v*≈165–195 cm^−1^ suggests that the carboxylate groups could be forming either bridging or bidentate complexes with the Mn-OH functional groups on the birnessite surface, in a ligand exchange complexation reaction^31^,^32^,^33^,^34^. Other authors have found that carboxylate can complex amorphous manganese oxides (poorly crystalline hexagonal birnessites) have a Δ*v*≈220 (refs 26, 36) and also assigned to the formation of either bridging or bidentate carboxylate complexes.

The broad band around 3,200 cm^−1^ present in all the spectra corresponds to the stretching of O-H (ν_OH_) and can be attributed to the presence of hydroxides, alcohols or, more likely, water. The bands observed around 2,550 cm^−1^ (ref. 37) are most probably caused by linearly adsorbed CO_2_, where the molecules interact via one oxygen atom with metal cations^38^.

To explore any redox reactions between the sequestered OC and birnessite we performed fluorescence spectroscopy (Table 1) on the original DOC molecules (from the peaty uplands of the WTW catchment) and on the soluble fraction of the surface Mn-OC, which was released into de-ionized water (amended to pH 5 like the original peaty water DOC) after reaction with the birnessite surface, called Mn-OC_DOC_. Both the original DOC and Mn-OC_DOC_ have a large fraction of fulvic-like material, with peak A^39^ (fulvic-like fluorophore of intensity 30) twice the intensity of peak C^39^ (humic-like fluorophore of intensity 16) (see Table 1).

There is a blue shift in the emission wavelength from original peaty water DOC to Mn-OC_DOC_ in both the Peak A (41 nm) and Peak C (40 nm) fluorophores^39^. This suggests that the reaction of the birnessite with the original DOC results in the release of transformed organic molecules, Mn-OC_DOC_, which have a lower aromaticity than the original DOC for both Peak A and Peak C^39^. This was anticipated as Mn oxides are capable of transforming HMW organic molecules into LMW molecules via oxidative transformations, which involves breaking aromatic carbon-carbon bonds^6^.

Taken together, the FTIR spectroscopy and fluorescence spectroscopy data suggest that the DOC is strongly adsorbed to the birnessite via carboxylate functional groups, and that, as a result of the adsorption reaction, the DOC is at least partially transformed into more aliphatic molecules.

### Bulk Mn-OC via TGA and XPS analysis

**Figure 3:**
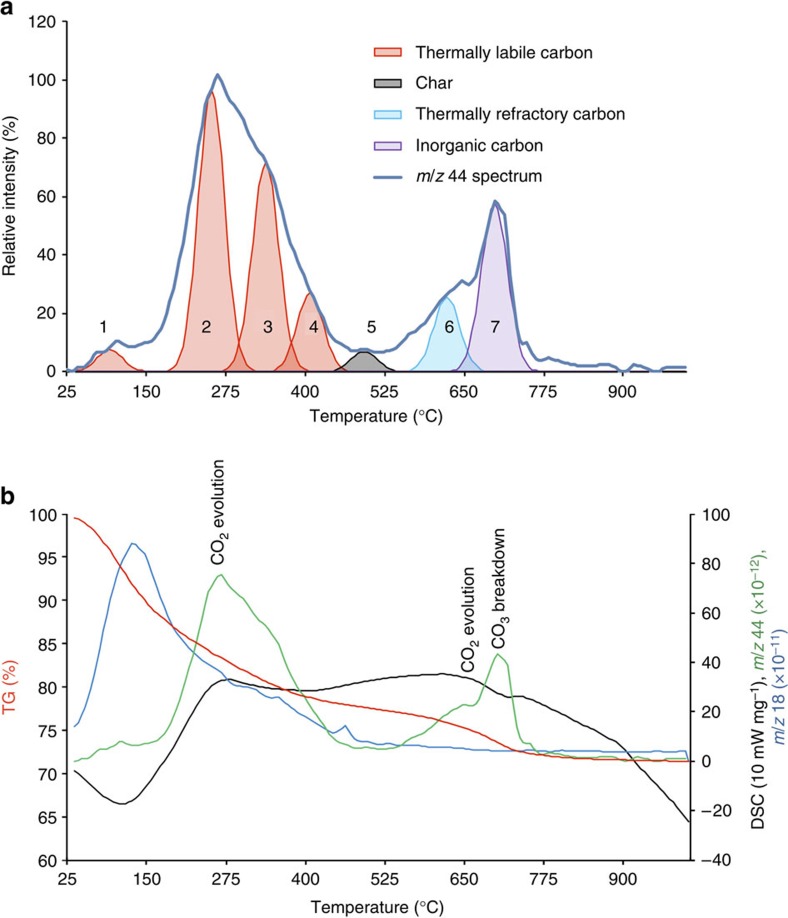
TGA-DSC data on birnessite coating. (**a**) Carbon dioxide evolution with temperature and synthesized deconvolution components (see Methods section for more details) for bulk Mn-OC showing thermally labile Mn-OC (in pink), thermally refractory Mn-OC (in blue) and inorganic carbon (in purple; TIC is 0.5%w/wC). (**b**) Differential scanning calorimetry (black) curve showing labelled CO_2_ evolution at 270 and 600 °C (exotherms) and CO_3_ ^2−^ breakdown at 712 °C (endotherm); *m/z* 44 curve (green); *m/z* 18 curve (blue) and thermogravimetric weight loss (red).

To gain a better understanding on the kinetic properties of the Mn-OC in the birnessite coating we next investigated the thermal reactivity of the carbon using TGA. The partitioning of carbon between organic (OC, 3.0%w/wC) and inorganic (IC, 0.5%w/wC) pools based on the TGA (up to 800 °C) of the bulk material is shown in Fig. 3a as the *m/z* 44 (CO_2_ and N_2_O—see Methods section) trace. Carbon dioxide (*m/z* 44) is detected at 200–375 °C (associated with thermally labile OC) and 550–750 °C (associated with thermally refractory OC). The thermally labile fraction is represented by the pink peak components (labelled nos. 2–4) and represents ∼89% of the Mn-OC, which is available to 800 °C. The thermally refractory fraction is represented by the blue peak component (labelled no. 6) and represents ∼11% of the Mn-OC, which is available to 800 °C. Inorganic carbon (IC) as calcite, with no interference by other decomposition reactions, gives a weight loss at around 720 °C. The purple peak (labelled no. 7) present at 712 °C is assigned to IC breakdown. This is corroborated by an endothermic reaction at 712 °C, visible in the differential scanning calorimetry (DSC) curve (see Fig. 3b).

Figure 3b shows that water (*m/z* 18) is detected at 25–200 °C and 200–400 °C and constitutes the largest proportion of the overall weight loss. Water loss is associated with surface dehydration and mineralogical transformation of birnessite^40^,^41^ and decomposition of OC^42^,^43^. There is limited available data on the TGA of synthetic birnessite^44^,^45^ but more detailed studies on other Mn oxides including manganite (MnOOH)^40^ and γMnO_2_ (ref. 41) show a progressive weight loss from 200 to 580 °C, which is due to water and some oxygen loss, with a sudden and significant weight loss at 620 °C, assigned to a mineralogical transformation to bixybite, Mn_2_O_3_ (refs 40, 41, 44). The DSC curve shows exothermic reactions at 270 and 600 °C , which could be associated with the breakdown of labile and refractory OC, respectively. The exothermic reaction at 600 °C is very near the temperature (620 °C) where the mineralogical transformation of Mn oxides to bixybite occurs^40^,^41^,^44^. A change in mineralogy at 620 °C may therefore explain some of the release of the thermally refractory Mn-OC suggesting that physical encapsulation may be a factor in determining how Mn-OC is sequestered and when Mn-OC is released.

Deconvolution (see Methods section) of the TGA data (Fig. 3a) reveals that thermally labile Mn-OC consists of at least three peak components, potentially representing three types of OC, which have previously been assigned as broadly cellulose-like (peak 2), lignin-like (peak 3) and more polycondensed material (peak 4)^42^ with cellulosic-like carbon dominating. The small peak present at 500 °C (peak 5) is typically associated with *in situ* generation of a char, which is an artefact produced as an intermediate product from the thermal decomposition of thermally labile-cellulosic-like material^46^. Leifeld^46^ examined the potential for discriminating between two types of thermally refractory OC: first, one which is inherently present in soils and sediments, and second, one which is likely produced as an artefact of the heating process (char). Leifeld^46^ concluded that exotherms derived at 520 °C or higher are derived exclusively from thermally refractory OC that is inherently present in the sample and not from artefact chars. We are therefore confident that the exotherm, which we identify at 600 °C in the bulk birnessite coating, represents material that is inherently present in the coating and not an artefact of burning.

**Figure 4:**
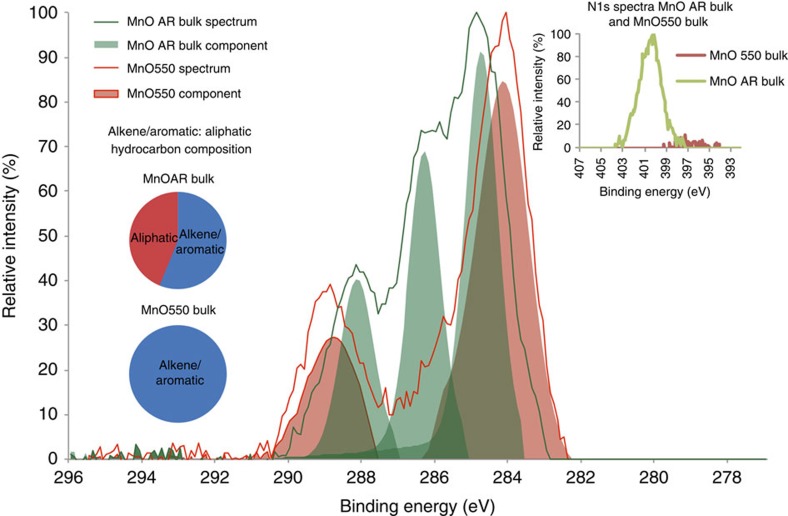
XPS C1s data on thermally treated bulk birnessite coating. Normalized and mean (*n*=9) XPS scans of the high-resolution C1s spectra of bulk birnessite coating both as received (MnOAR Bulk) and thermally treated by burning to 550 °C for 8 h (MnO550 Bulk), de-convoluted using synthetic component fitting (see Methods section). There are three main components evident in the bulk birnessite coating, and there are two main components evident in the bulk birnessite coating that has been burned to 550 °C. The most probable chemical assignments for these components are described in Table 2. The pie chart shows the proportion of hydrocarbon that is aromatic or aliphatic in character in each of the ‘as received' and ‘thermally treated' bulk coating. The inset shows N1s scan. Note ‘aromatic' in pie chart actually represents alkene/aromatic peak at ∼285 eV.

The peak at 600 °C representing thermally refractory OC has not previously been reported in the literature as soil organic matter or mineral-associated organic carbon^47^, but is usually reported as black carbon^12^,^13^. The peatlands in the WTW catchment are occasionally burned and so particulate black carbon originating from the peatlands^44^ may be present in the influent water on occasion. However, peat-derived black carbons usually contain detectable N^48^ and the refractory Mn-OC contains no detectable N (see Fig. 4 inset). For this reason it seems more likely that the refractory Mn-OC in our samples is not black carbon but organic matter that is adsorbed to the birnessite and that adsorption to the birnessite surface is the reason why the OC is decomposed more slowly and to a lesser extent than uncomplexed OC^3^.

To understand the difference in chemical state between the thermally labile and thermally refractory Mn-OC, XPS analysis of the bulk birnessite coating as received (MnOAR Bulk) and the bulk birnessite coating thermally treated to 550 °C (MnO550 Bulk) was undertaken (see Fig. 4). The Mn-OC (available up to 800 °C) in the MnOAR bulk samples contains both 89% thermally labile and 11% thermally refractory Mn-OC; deconvolution of the XPS data (see Methods section) reveals four components consistent with alkene/aromatic (284.9 eV), aliphatic (286.5 eV), amide (∼288 eV, but this peak occurs at the same binding energy as the carboxyl peak component) and carboxyl (288.3 eV) bonds (see Table 2; Fig. 4). In contrast, the thermally refractory Mn-OC (MnO550 Bulk and MnO1000 Bulk, which is the bulk material thermally treated to 550 and 1,000 °C, respectively) contains alkene/aromatic and carbonyl (carbonate and carboxylate) groups only (Table 2; Fig. 4). It should be noted that, in MnO1000 Bulk, the carbonyl compounds are ∼60% reduced (*cf*. MnO550 Bulk) and consist of only carboxylate compounds, since the carbonate fraction is decomposed at 712 °C (see Supplementary Fig. 1). This was also confirmed with a high resolution Ca 1s spectra (see Supplementary Fig. 2). The XPS data therefore confirm that the thermally labile and thermally refractory Mn-OC pools are chemically distinct, with the thermally refractory Mn-OC (represented by MnO550) being depleted in N-rich compounds (as shown in the N1s scan inset in Fig. 4).

The bulk birnessite coating as received (MnOAR Bulk) has a C:N ratio of 15:1, which is much lower than the original DOC C:N ratio of 60:1 (ref. 49). This may suggest that selective preservation of N-rich organic material is occurring during the initial adsorption of DOC onto birnessite. A small amount of the N in the Mn-OC may be due to selective adsorption of a cationic flocculant used, but this is likely to be a very small component (see Methods). High N concentrations are also present in the Fe-OC of marine sediments^4^. However, this N-rich material is not detectable in the thermally refractory Mn-OC fraction (represented by MnO550 Bulk in Table 2 and inset N1s scan in Fig. 4). As the thermally refractory OC is depleted in N, we argue that it cannot have been formed via the polyphenol-Maillard reaction^23^ as this type of polymerization reaction involves condensation between amino acids (which contain N) and polyphenols and/or sugars.

**Figure 5:**
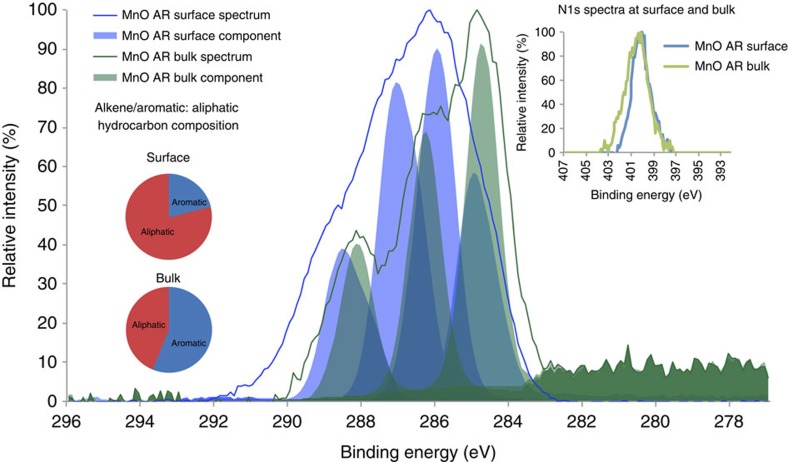
XPS C1s data comparing surface and bulk birnessite coating. Normalized and mean (*n*=9) XPS scans of the high resolution C1s spectra of bulk birnessite coating (MnOAR Bulk) and the surface birnessite coating (MnOAR Surface, intact on the sand grain), both as received, de-convoluted using synthetic component fitting (see Methods section). There are three main components evident in the bulk birnessite coating, and there are four main components evident in the surface birnessite coating. The most probable chemical assignments for these components are described in Table 2. The pie chart shows the proportion of hydrocarbon that is aromatic or aliphatic in character in each of the ‘as received bulk' and ‘as received surface' coating. Note ‘aromatic' in pie chart represents alkene/aromatic peak at ∼285 eV and mean components are not Gaussian. Inset shows N1s scan.

The Mn-OC bound at the surface of the birnessite coatings has a significantly lower ratio of alkene/aromatic:aliphatic hydrocarbons (1:4) compared with the Mn-OC present throughout the bulk birnessite coatings (3:2) (see pie-charts in Fig. 5).

The bulk birnessite coating as received (MnOAR Bulk) has a δ^13^C signature of ∼−24.9‰ (*n*=3, s.d.=0.1), which is statistically significantly less depleted in δ^13^C compared with the influent water (∼−27.0‰). Lalonde *et al.*^4^ also found a less depleted δ^13^C signature in Fe-OC in marine sediments, which they attributed to selective sorption of an isotopically less depleted fraction of OC by mineral phases. Interestingly, the bulk birnessite coating that has been thermally treated to 550 °C (MnO550 Bulk, containing only thermally refractory Mn-OC) has a δ^13^C signature of ∼−27.6‰ (*n*=3, s.d.=0.3) that is statistically significantly different from the bulk material as received (*P*<0.01) but not statistically significantly different from the influent water signature (∼−27.0‰).

### Proposed carbon sequestration and stabilization mechanism

**Figure 6:**
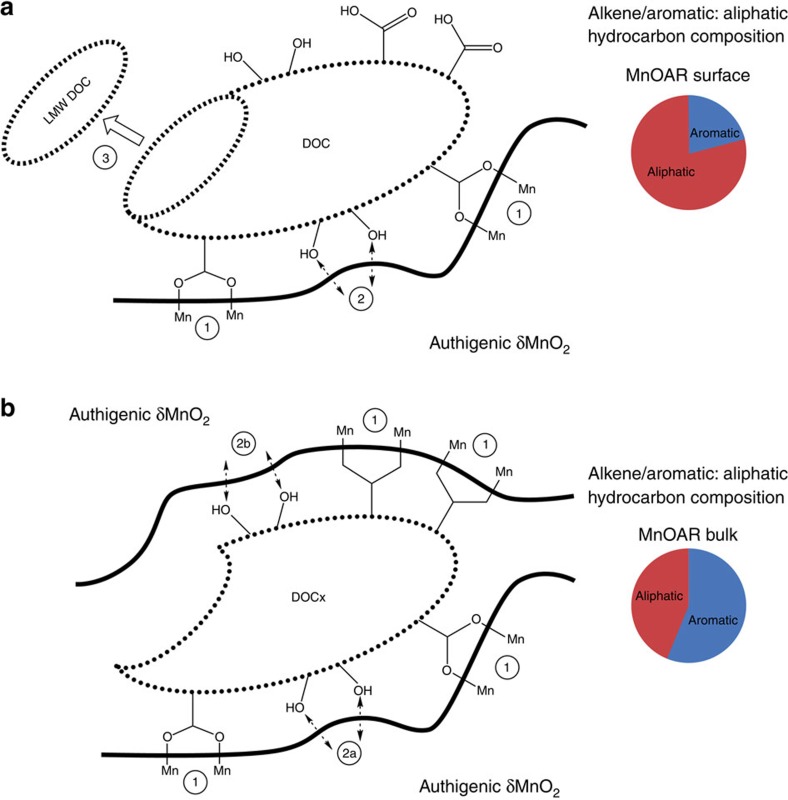
Hypothesized reaction mechanism between DOC and birnessite. Hypothesized reaction mechanism for conversion of DOC by birnessite coatings both at the surface ((**a**) points 1–3) and in the bulk material ((**b**) points 1–2). The dashed arrows represent phenol–birnessite interactions resulting in phenoxy radical formation^52^. The original DOC molecule is oxidized (via the breaking of aromatic bonds^6^) at point 3 resulting in the release of LMW molecules into solution as the birnessite surface is subjected to backwashing in the WTW. The alkene/aromatic:aliphatic ratio (XPS signature) of the resulting smaller molecule DOCx attached to the birnessite surface is shown in pie-chart **a**. Once DOCx becomes trapped (shown in (**b**)) the reactions between phenols and the birnessite surface are likely to result in a build up of phenoxy radicals as these are less likely to be washed away at depth in the bulk material. The notably different XPS signature of the bulk material is shown in pie-chart (**b**).

**Figure 7:**
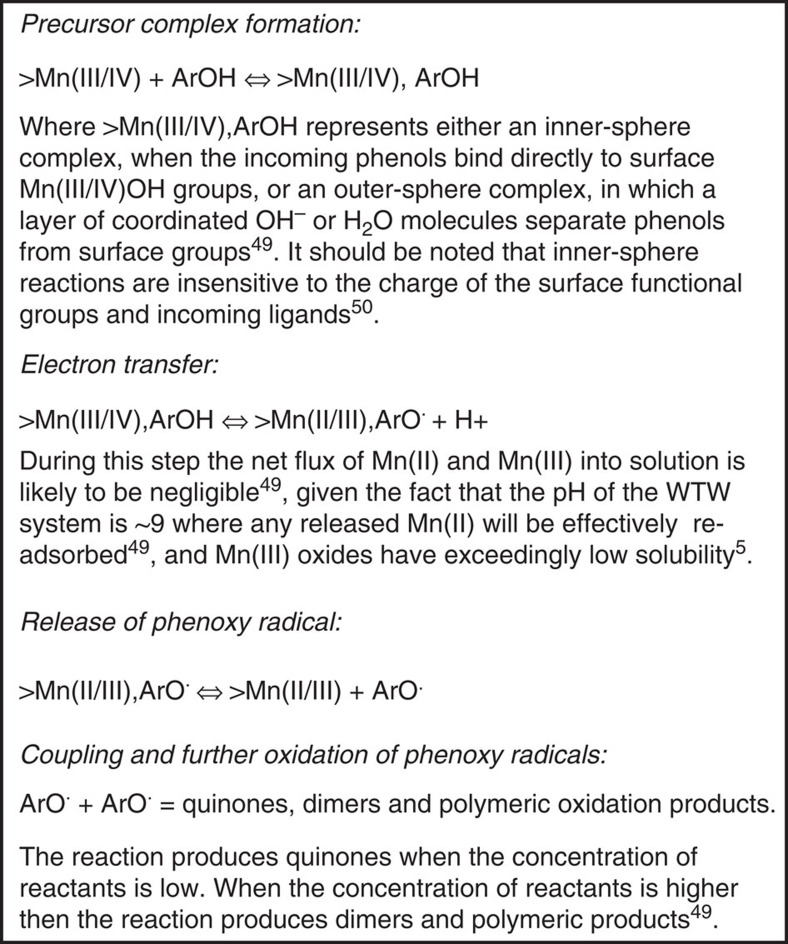
Reaction scheme between birnessite and phenol groups. Surface complex formation between birnessite and phenol groups in DOC^52^. In the above reaction scheme the protonation state of the birnessite surface functional groups (as Mn(III/IV)OH_2_^+2/3^ or MnOH(III/IV)^−1/3^), the phenol ion (as ArOH or ArO^−^) and reaction intermediates are ignored. However, because the pK_a_ of poorly crystalline hexagonal birnessite is ∼2 (ref. 26), while the pK_a_ of most phenolic –OH groups is greater than 9 (ref. 52), then at pH∼9 in the WTW system, the majority of the birnessite surface groups will be present as negatively charged Mn(III/IV)OH^−1/3^, while phenol ions will be present as both ArOH or ArO^−^.

Our data show for the first time that Mn-OC is adsorbed to the surface of birnessite (and trapped within the mineral) via a strong manganese carboxylate bonds (Fig. 6, point 1). We propose that the DOC molecule is tethered, via carboxylate bonding (Fig. 6, point 1). This allows DOC molecules that contain abundant phenol groups^50^ to react with the birnessite surface (Fig. 6, point 2a). Phenols are known to be a redox mediator^51^, capable of catalysing redox reactions, but more importantly they also react with birnessite to form phenoxy radicals via the reactions shown in Fig. 7 (ref. 52).

Phenoxy radicals are then either converted to quinones, or encounters between phenoxy radicals result in radical coupling to produce dimers or polymeric products^52^. Stone^52^ suggests that when the concentration of reactants is low then phenoxy radicals tend to form quinones and that when the concentration of reactants is high, phenoxy radicals will tend to form dimers and polymeric products. Owing to the frequent backwashing in the filter beds it seems likely that the concentration of phenoxy radical containing products at the surface will be kept low as reactants and products are constantly being removed from the surface. We speculate that quinone formation is the most likely reaction pathway occurring between birnessite and DOC molecules at the outermost surface of the birnessite coatings (Fig. 6a). During the course of these reactions, HMW organics are converted to LMW organics (see Fig. 6, point 3) by breaking aromatic bonds^6^, and thus the original DOC molecule is transformed into DOCx, creating the alkene/aromatic: aliphatic ratio of 1:4 that we measure in the surface-bound Mn-OC.

When the next layer of birnessite precipitates, DOCx is then trapped and the uppermost surface of the DOCx molecule can adsorb to the new birnessite surface via the manganese carboxylate bonds (also labelled point 1 in Fig. 6), instigating further phenolic–birnessite interactions (labelled as point 2b on Fig. 6b). If the redox reactions between phenol groups and birnessite occurring at depth within the bulk material were the same as at the surface then the ratio of alkene/aromatic: aliphatic ratio should theoretically continue to decrease, as more redox reactions occur breaking up more aromatic bonds. However, the ratio of alkene/aromatic:aliphatic actually increases significantly from 1:4 in surface-bound Mn-OC to 3:2 in the bulk Mn-OC. We do not know the reason for this stark change in carbon chemistry. The main difference in the reaction environment is that at the surface of the birnessite any reaction products formed could be removed by backwashing, whereas in the bulk material, which is not exposed to backwashing, phenol–birnessite interactions are more likely to lead to a build-up of phenoxy radical containing products that could lead to dimer formation and polymeric products^52^. However, the mechanisms presented in Fig. 7 are likely to occur as a continuum between surface and at depth within the bulk material.

Polymerization of phenoxy radicals can create C-C bonds and C-O bonds linking benzene rings^53^. These bonds are aliphatic suggesting polymerization should result in an XPS signature with a lower alkene/aromatic: aliphatic ratio. However, our XPS data show a significant increase in the alkene/aromatic: aliphatic ratio. There has been much work carried out on the cross-coupling of phenolic molecules with humic substances using NMR^53^ but no published literature as far as the authors are aware on the XPS signature of such linkages. It is possible that C-C bonds that link benzene rings could lead to an increased alkene/aromatic signal due to the more polymerized nature of the products but we have no evidence for this. The XPS data do not contradict the formation of C-O (ester or ether bonds) either, but, because the carbonyl bond occur at binding energies that overlap with ether bonds, XPS cannot discriminate between the C-O bonds. We are not suggesting that the thermally refractory nature of the Mn-OC is due to polymerization, we only invoke this reaction pathway to explain the stark change in alkene/aromatic:aliphatic ratio between surface-bound Mn-OC and bulk Mn-OC.

We observe with TEM 7 and 10 Å birnessite in the Mn oxide coating (see Fig. 1). We know that 10 Å birnessite can form from 7 Å birnessite as a secondary precipitate during dissolution-recrystallisation reactions under diagenesis^54^. We also know that physical entrapment of C can also cause 10 Å birnessite to form from 7 Å birnessite^55^. The presence of 10 Å birnessite in our system is therefore consistent with the diagenetically driven redox reactions and physical entrapment of OC that we postulate.

## Discussion

Clearly the WTW presents environmental conditions different from most natural terrestrial and marine environments. Indeed it is precisely this controlled environment that has allowed us to gain a fundamental understanding of the underlying reaction mechanisms that are responsible for the sequestration and stabilization of organic carbon by birnessite. To evaluate what role natural birnessites play in sequestering and transforming carbon on a global scale, however, more research is required, specifically to determine whether the components of the WTW system are similar enough to natural environments to provide a representative analogue.

While we identify poorly crystalline hexagonal birnessite in the WTW, which is one of the most common Mn oxide minerals present in terrestrial^18^,^19^ and marine systems^20^,^21^,^22^, there are potentially important differences between the WTW birnessite and that found in natural environments. This could hint to additional reaction mechanisms, and affect the reaction rate, of organic carbon sequestration and transformation. For example, differences in the pH at which birnessite is precipitated (where the WTW birnessite is precipitating at a higher pH (pH 9) than in most natural environments (rivers typically being ∼pH 6–8 and seawater ∼pH 8)) and/or the influence of Mn(II) oxidation by microorganisms (where the WTW birnessite is likely precipitated via the abiotic oxidation of Mn(II/III), while it is known that microorganisms can significantly enhance Mn oxidation in natural systems^56^) can result in subtle structural differences between different poorly crystalline hexagonal birnessites^57^, which could influence the rate at which organic carbon is sequestered and stabilized.

Importantly, having a controlled system has allowed us to identify a fundamental mechanism for how birnessite can trap and stabilize organic carbon. Specifically, we have determined that carboxylate groups are involved in bridging and/or bidentate bonds that bind DOC molecules to birnessite, both at the mineral surface and within the bulk mineral, and that these bonds at least partly withstand washing with 1 M NaOH solution. We conclude that this bonding of DOC by carboxylate groups, in conjunction with the physical trapping of DOC within the ‘onion shell' layers of the birnessite coating, is responsible for the thermally refractory nature of 11% of the Mn-OC (that is Mn-OC which is available to 800 °C). Further research is required to confirm whether OC is physically trapped in birnessite found in the natural environment.

Furthermore, we propose that phenol groups within the DOC molecule are oxidatively transformed to phenoxy radicals, following the well-known birnessite-phenol reaction pathway^52^, leading to a continuum of reactions from surface to bulk material, including the production of LMW organic molecules (with potential complete mineralization to CO_2_ and H_2_O) and the formation of polymerized products in the bulk material where the concentration of phenoxy radical products can build up^52^. Whether birnessite can catalyse the polymerization of sequestered organic carbon, and how this process might affect the fate of buried organic carbon in natural systems is not known and requires further research. However, as birnessite is effectively recycled at oxic-anoxic redox interfaces^58^, the strong association between Mn and C may not be preserved in the deeper geological record^8^. It is therefore important to note that only a spectrum of studies looking at both controlled and natural systems will allow us to fully understand the relation between OC and metal (hydr)oxides.

We suggest that this initial mechanistic understanding of the role of birnessite in carbon stabilization may help in our understanding of the global carbon cycle.

## Methods

### Water treatment works

The birnessite-coated sands come from Mosswood Water Treatment Works (WTW) in the UK (54°51°N-53°59°W). Mosswood is a Mn-removing WTW (owned by Northumbrian Water Ltd, UK) that employs chemical oxidation and filtration to remove dissolved Mn(II) from potable water. Such WTWs are prevalent globally in temperate regions where influent water contains dissolved Mn and DOC, which are both problematic for the water industry for aesthetic reasons^59^. Soluble Mn(II) undergoes sorption to filter sand surfaces followed by oxidation to insoluble manganese oxides utilizing the autocatalytic nature of manganese oxidation at pH9. Some anthracite is also used as an additional filter media. ^14^C analysis was used to confirm that the bulk Mn-OC is 100% modern organic matter to ensure that there was no interference from the anthracite.

Northumbrian Water Ltd. confirmed that these sands were in place for ∼40 years. Replacement of the filter media usually takes place due to drop in Mn removal efficiency either due to lack of porosity or an unknown cause. The influent water at Mosswood (132.5 Ml per day) originates from the Pennines peat uplands and influent water TOC is ∼10 mg l^−1^ present as both particulate organic carbon (POC or suspended solids) and dissolved organic carbon (DOC). Dissolved Mn is ∼0.2 mg l^−1^. Ferric sulphate is added as a coagulant to the influent water at the WTW to remove the majority of suspended solids (both organic and inorganic particulates) in the secondary treatment process before the water enters the Mn filter beds. A flocculant (a modified starch with cationic amine groups) is also added at very low dosage rates (*cf* natural DOC which is >1,000 kg day^−1^) in the secondary treatment process and most of this is removed as flocculated solids known as water treatment residual. Any flocculant remaining in solution could enter the Mn filter beds but the ratio of influent DOC to flocculant is likely to be high and we assume that the flocculant does not constitute a large part of the Mn-OC.

The birnessite-coated sand was analysed in two fractions, at the surface of the intact coating and at depth that was represented by the bulk material separated from the sand grain. The 63–149 μm fraction of the birnessite coating (captured from sieved intact sand grains by shaking) was (arbitrarily) used to represent the bulk material. All the other size fractions also contained ∼3%w/wC. TC, TIC, TOC and N were determined using a LECO Elemental analyser. Organic carbon (carbonate-free) isotopic abundances were analysed by IsoAnalytical Ltd (Crewe, UK), using a Europa Scientific 20:20 instrument (up to 1,700 °C). Results were recorded relative to the Vienna Peedee Belemnite standard (v-PDB). Samples were acid washed with 2 M hydrochloric acid and analysed by elemental analyser-isotope ratio mass spectrometry. Replication had a s.d. better than ±0.1‰ and values for reference materials were within two s.d. of the expected result. ^14^C analysis was carried out at SUERC (Glasgow University) to confirm that Mn-OC was not anthracite.

### Thermogravimetric analysis

Thermal analysis was carried out to determine the relative proportions of different C components according to the method of Lopez-Capel *et al.*^43^, extended by determination of evolved gases. A Netzsch STA 449C Jupiter TG-DSC system connected by a heated capillary (200 °C) to a Netzsch Aeolos quadrupole mass spectrometer system was used to determine the following parameters: (1) mass change, (2) energy gained or lost from the sample, (3) composition of evolved gases (*m/z* range 10–300). Individual powder samples (typically 10 mg) were placed within an alumina crucible, using an identical empty reference crucible, and were heated from ambient temperature to 990 °C under flowing He80-O20. Intensities for *m/z* 44 (CO_2_ +N_2_O) and *m/z* 18 (H_2_O) were recorded. No correction was made to *m/z* 44 for evolved N_2_O, which was assumed to be negligible compared with CO_2_ given failure to detect measurable *m/z* 30 (NO). Evolved gas evolution curves were interpreted using GRAMS/AI peak fitting software (www.AdeptScience.co.uk) that permits investigation of the contribution of several individual peaks to an overall trace. By defining initial conditions (including a presumed Gaussian peak shape), the range of potential values for each peak centre (25–200 °C, 200–375 °C, 375–550 °C and 550–800 °C respectively) and the number of iterations to perform (in this case 50), the software fits individual labile, recalcitrant and refractory carbon peaks^60^ such that their combined shapes and areas are the best possible fit for the overall CO_2_ trace.

Thermal treatment (to generate MnO500 and MnO1000 samples) was conducted by placing 2 g sample in a ceramic thimble then heated for 8 h in a 6 litre Carbolite ELF muffle furnace and allowed to cool to ambient temperature before being placed in 10 ml glass vials and sealed with Al foil between the screw caps for storage.

### Focused ion beam, scanning electron and transmission microscopy

FIB cross-sections were performed using FEI Helios Nano Lab 600 Dual Beam system, equipped with focused Ga liquid metal ion source. Ion beam thinning carried out at 52° with respect to electron column. Initially a ‘rough cut' was carried out after depositing a protective platinum layer *in situ* using a gas injection system. Several *in situ* polishing steps were performed on exposed cross-sections to produce a clean surface with minimal beam damage. High-resolution backscattered electron imaging was carried out in the same system, with images being captured using a low kV immersion lens.

SEM imaging was performed on a Hitachi SU70 high-resolution analytical SEM, equipped with Oxford instruments INCA energy 450 and WDS 700. The JEOL 2100F transmission electron microscope was operated at 80 KeV.

Micro-FTIR analyses were carried out using a Perkin Elmer Spotlight FT-IR imaging system. Reflectance micro Fourier Transmitted Infrared spectra were collected over the 4,000 to 700 cm^−1^ wavenumber range, at a resolution of 4 cm^−1^, beam diameter 15 μm.

### X-Ray photoelectron spectroscopy

XPS spectra were obtained at the National EPSRC XPS Users' Service (NEXUS) at Newcastle University. 0.5 grams of sample was immobilized onto a Si wafer using 3 M double-sided adhesive. The immobilized samples were decontaminated within the instrument by sputter etching, using a Ag^+^ gas cluster ion beam (GCIB) gun at 4 kv for 30 s. The Ag^+^GCIB sputter etching was carried out in polyatomic mode to avoid degrading the chemistry during the etching process. The samples were then analysed using a Thermoscientific K-alpha X-ray photoelectron spectrometer (XPS) (Thermoscientific, East Grinstead, UK).

Low-resolution full spectra were acquired using a monochromatic Al Kα X-ray source with an output energy of 1,486.6 eV. An X-ray beam spot size of 50 μm, and a dwell time of 100 ms was used for both high resolution and survey spectra. The surface charge compensation was achieved using a low-energy electron flood gun, which was operated at 40 eV. Survey spectra were obtained between a binding energy of 0 and 1,350 eV with a 1.0 eV step size and pass energy of 200 eV.

Analysis of the survey spectra was carried out using CasaXPS software (Teighmouth, UK), and major peaks were selected for element identification using the Handbook of X-ray Photoelectron Spectroscopy^61^. The major peaks for carbon and nitrogen were selected for high-resolution scans from the survey spectra. C1s high-resolution spectra were obtained from binding energies of 279.0 to 298.0 eV and N1s high-resolution spectra between 410.0 and 392.0 eV. The step size for the high-resolution spectra was 0.1 eV with a 40 eV pass energy. Shirley backgrounds were applied to the high-resolution spectra and the minimum number of synthetic Gaussian components were fitted to the C1s high-resolution scans to elucidate the chemical states of the carbon using the CasaXPS software algorithm. The NIST (srdata.nist.gov/xps) and LA surface (lasurface.com) databases were used for component peak assignment in conjunction with other references^61^,^62^.

The spectral data of the peaks, their components and their backgrounds, from each of the replicates, were acquired from the C1s and the N1s high-resolution scans. These were obtained from the surface fraction, the bulk fraction and the 550 and 1,000 °C thermally treated samples. The digital CasaXPS spectral data were transferred to Microsoft Excel 2010. The mean spectral intensity of replicates and their components were calculated and the mean Shirley backgrounds were deducted. The mean data were then normalized to the highest spectral intensity. The mean normalized spectra and their normalized mean components were then plotted against binding energy.

### Data availability

The data presented in this paper are available from http://dx.doi.org/10.15128/br86b364d.

## Additional information

**How to cite this article:** Johnson, K. *et al.* Towards a mechanistic understanding of carbon stabilization in manganese oxides. *Nat. Commun.* 6:7628 doi: 10.1038/ncomms8628 (2015).

## Supplementary Material

Supplementary Information Supplementary Figures 1-2

## Figures and Tables

**Table 1:** Fluorescence spectroscopy data for DOC and Mn-OC_DOC_.

**All at pH5**	**Peak A—‘fulvic-like'**	**Peak C—‘humic-like'**
DOC (from peaty water)	223:454 (30)	342:450 (16)
Mn-OC_DOC_	213:413 (30)	296:410 (16)

DOC, dissolved organic carbon; Excitation:emission wavelengths (nm, with maximum intensity in adsorption units in brackets) of Peak A (fulvic-like) and Peak C (humic-like) for both the original peaty water DOC and the Mn-OC_DOC_. *n*=3 and s.d. values <2 nm and <0.5 adsorption units.

**Table 2:** XPS C1s data for birnessite coating.

**Component positions (eV)**				**Chemical state**	**Principal assignments**
**MnO AR Surface**	**MnO AR Bulk**	**MnO550 Bulk**	MnO1000 Bulk*		
285.2	284.9	284.3	284.3	C(=C,-H)	Alkene and aromatic
286.1	286.5	ND	ND	C(-C,-H,-N)	Aliphatic†
287.2	Peak hidden‡	ND	ND	C(-N,-O)	Amides‡
288.6	288.3	288.9	288.9§	C(-O,=O)	Carboxylate and CO_3_ ^2−^

ND, not determined; The mean (*n*=9) binding energy positions of the synthetic components, fitted to C1s spectra. The potential chemical state assignments for these components for MnOAR Bulk (bulk birnessite coating as received), MnO550 Bulk (bulk birnessite coating thermally treated to 550 °C) and MnO1000 Bulk (bulk birnessite coating thermally treated to 1,000 °C) are based on multiple lines of evidence, obtained from high-resolution C1s (shown above and in Figs 4 and 5 and Supplementary Fig. 1) and N1s (inset in Fig. 4) and Ca 2p scans (see Supplementary Fig. 2), and FTIR spectroscopy (see Fig. 2). The bonding environment in all the samples is more complex than represented here, and the complex chemical environment will contain many overlapping Gaussian components. To simplify the deconvolution analysis, the minimum number of components were fitted to the scans and the component assignment interpretation represents the principal chemical state (see Methods section). The changes in component positions between the MnOAR and thermally treated MnO550 and MnO1000 are primarily the result of the removal of thermally labile organic material during thermal treatment.

^*^Data not presented in Fig. 4 (see Supplementary Fig. 1)

^†^Thermal treatment to 550 °C that removes N (N1s data presented as inset in Fig. 4) suggests a C-N contribution to this component

^‡^FTIR spectroscopy and N1s high-resolution scan supports amide rather than C-O interpretation

^§^Ca2p high-resolution scans (see Supplementary Fig. 2) and decomposition of carbonate at 712 °C (see Fig. 3) supports a carboxylate rather than a carbonate interpretation.
