# Supplementary material for: Towards a mechanistic understanding of carbon stabilization in manganese oxides
Source: Nat Commun. 2015 Jul 21;6:7628. doi: 10.1038/ncomms8628 (PMC4518293; doi:10.1038/ncomms8628)
Supplement: Supplementary Information — Supplementary Figures 1-2 [file ncomms8628-s1.pdf]

X-ray photoelectron spectroscopy (XPS) analysis

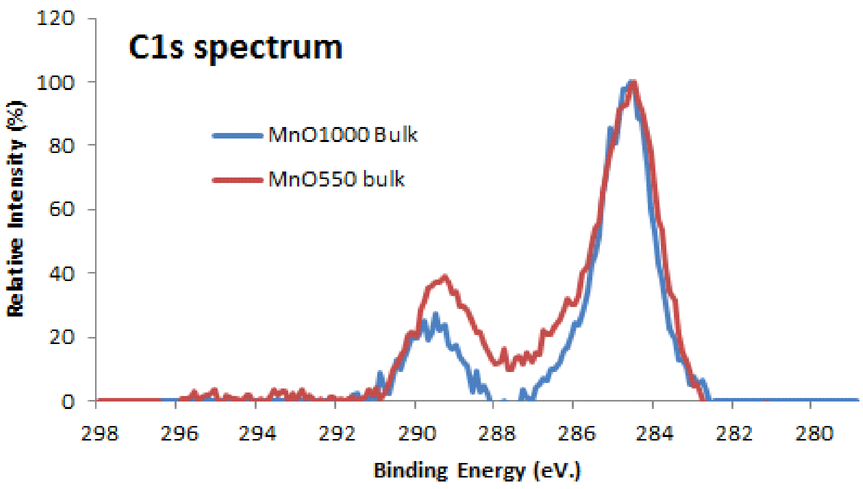

Supplementary Figure 1: Normalised and mean (n=3) XPS scans of the high resolution C1s spectra for thermally treated (1000°C for 8 hours) bulk birnessite coating MnO1000 and normalised and mean (n=9) XPS scans of the high resolution C1s spectra for thermally treated (550°C for 8 hours) bulk birnessite coating MnO550

Supplementary Fig. 1 shows the XPS data for the C1s high resolution scan for the birnessite coating thermally treated to 1000°C for 8 hours (MnO1000 Bulk) and the birnessite coating thermally treated to 550°C for 8 hours (MnO550 Bulk). The two peaks represent carbon oxygen chemistry (288.9eV) and alkene/aromatic moieties (284.3eV), however it is clear that the 288.9eV peak in MnO1000 is reduced by ~60% cf to the MnO550 288.9eV peak which is consistent with the carbonate component of that peak undergoing thermal decomposition.

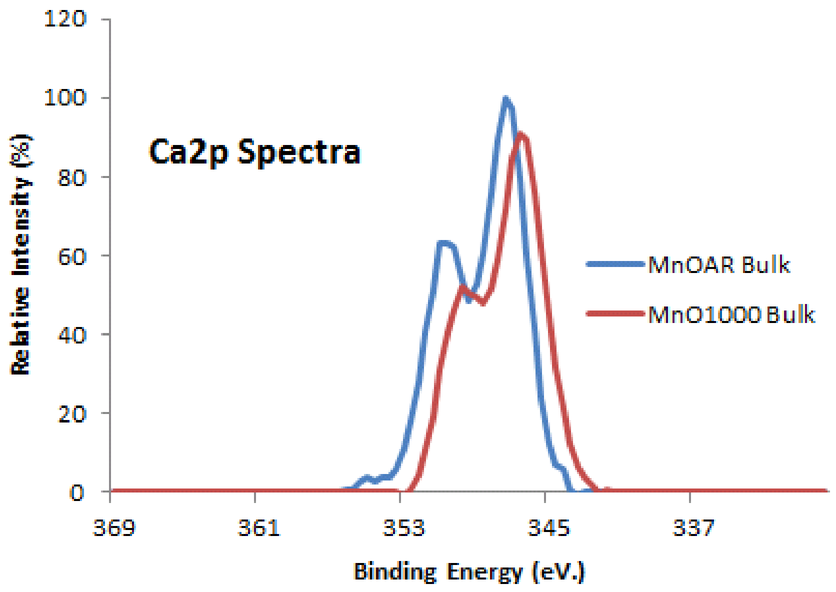

Supplementary Figure 2: Normalised and mean (n=3) MnOAR Bulk and MnO1000 XPS high resolution Ca2p scans (step size =0.4 eV) which illustrates the alteration in Ca chemistry due to thermal treatment. The region 351eV is consistent with the Ca2p1/2 orbital of $CaCO_3$ and the region from 350eV to 347eV is consistent with Ca2p3/2 orbital of CaO.

Supplementary Fig. 2 shows the xps data for the Ca2p high resolution scan for the bulk birnessite coating as received (MnOAR) and the birnessite coating thermally treated to 1000°C for 8 hours (MnO1000 Bulk). CaCO3 thermally decomposes in our birnessite coating at 712°C, and Figure 2 provides evidence that the xps spectra of MnOAR is significantly different to MnO1000, suggesting that the decomposition of CaCO3 has occurred, as a consequence of thermal treatment to 1000°C. This suggests that the reduction in the MnO1000 288.9eV peak to ~60% of 288.9eV peak in MnO500 is consistent with the loss of the carbonate component of this peak.
